# Identification of microRNAs differentially expressed in glioblastoma stem-like cells and their association with patient survival

**DOI:** 10.1038/s41598-018-20929-6

**Published:** 2018-02-12

**Authors:** Jiri Sana, Petr Busek, Pavel Fadrus, Andrej Besse, Lenka Radova, Marek Vecera, Stefan Reguli, Lucie Stollinova Sromova, Marek Hilser, Radim Lipina, Radek Lakomy, Leos Kren, Martin Smrcka, Aleksi Sedo, Ondrej Slaby

**Affiliations:** 1 Central European Institute of Technology (CEITEC), Masaryk University, Brno, Czech Republic; 2 Department of Comprehensive Cancer Care, Masaryk Memorial Cancer Institute, Faculty of Medicine, Masaryk University, Brno, Czech Republic; 3 Institute of Biochemistry and Experimental Oncology, First Faculty of Medicine, Charles University, Prague, Czech Republic; 4 Department of Neurosurgery, University Hospital Brno, Faculty of Medicine, Masaryk University, Brno, Czech Republic; 5 Department of Neurosurgery, University Hospital Ostrava, Ostrava, Czech Republic; 6 Department of Pathology, University Hospital Brno, Faculty of Medicine, Masaryk University, Brno, Czech Republic

## Abstract

Glioblastoma stem-like cells (GSCs) are critical for the aggressiveness and progression of glioblastoma (GBM) and contribute to its resistance to adjuvant treatment. MicroRNAs (miRNAs) are small, non-coding RNAs controlling gene expression at the post-transcriptional level, which are known to be important regulators of the stem-like features. Moreover, miRNAs have been previously proved to be promising diagnostic biomarkers in several cancers including GBM. Using global expression analysis of miRNAs in 10 paired *in-vitro* as well as *in-vivo* characterized primary GSC and non-stem glioblastoma cultures, we identified a miRNA signature associated with the stem-like phenotype in GBM. 51 most deregulated miRNAs classified the cell cultures into GSC and non-stem cell clusters and identified a subgroup of GSC cultures with more pronounced stem-cell characteristics. The importance of the identified miRNA signature was further supported by demonstrating that a Risk Score based on the expression of seven miRNAs overexpressed in GSC predicted overall survival in GBM patients in the TCGA dataset independently of the IDH1 status. In summary, we identified miRNAs differentially expressed in GSCs and described their association with GBM patient survival. We propose that these miRNAs participate on GSC features and could represent helpful prognostic markers and potential therapeutic targets in GBM.

## Introduction

Glioblastoma multiforme (GBM) is the most frequently occurring primary brain tumor of astrocytic origin in adults. Despite complex therapy consisting of maximal surgical resection, adjuvant concomitant chemoradiotherapy with temozolomide followed by temozolomide in monotherapy, the prognosis remains dismal^1^. The short survival of GBM patients is caused by both the impossibility of achieving “biologically” radical surgical resection and tumor resistance to adjuvant therapy. Glioblastoma stem-like cells (GSCs) are thought to be an important contributor to the poor response to the adjuvant therapy due to the higher expressions of the DNA repair enzymes, antiapoptotic factors, and multidrug transporters^2,3^. These rather slow proliferating cells are also capable of self -renewal and multilineage differentiation, are highly invasive, modulate immune response and promote angiogenesis. GSCs form gliomaspheres in serum-free media *in vitro*^4,5^ and have strong tumorigenic potential in immunodeficient animals recapitulating the hallmarks of the original tumors^6^. GSCs express, although to a variable extent, specific stemness markers such as the transcription factor Sox-2, the cytoskeletal protein nestin, and/or the cell surface glycoprotein CD133^7,8^, which are generally used for their identification. According to some studies, the presence of GSCs as determined by functional assays as well as the expression of GSC markers is associated with the prognosis in GBM patients^9–12^. Several studies have shown that microRNAs (miRNAs) are important molecular players closely related to the biological features of GSCs. MiRNAs are highly conserved, 18–25 nucleotide long non-coding RNAs that function as post-transcriptional regulators of gene expression by silencing their mRNA targets. It is estimated that miRNAs could regulate up to 60% of human genes including genes associated with the maintenance of the stem-like phenotype, differentiation, and chemo- and radioresistance^13,14^. Thus, miRNAs play significant roles in the functions of various types of healthy as well as cancer stem-like cells including GSCs^15–17^. Indeed, changes in miRNA expression were observed during the transition of GSCs to more differentiated phenotypes^18^ and e.g. the miR-302-367 cluster was shown to be able to abolish the stem cell characteristics of GSCs^19^. Our previous studies also demonstrated that miRNAs are able to predict the survival in GBM patients^20,21^.

In this study, we identified a set of miRNAs that is closely associated with the stem-like phenotype of GBM cells. We further corroborated the importance of the most differentially expressed miRNAs by showing their potential to predict overall survival in GBM patients independently of the IDH1 mutation status. These miRNAs may thus play an important role in the pathogenesis of brain tumors and represent potential therapeutic targets affecting GSCs and overcoming the therapeutic resistance of GBM.

## Results

### Characterization of the paired glioblastoma cell cultures

**Figure 1:**
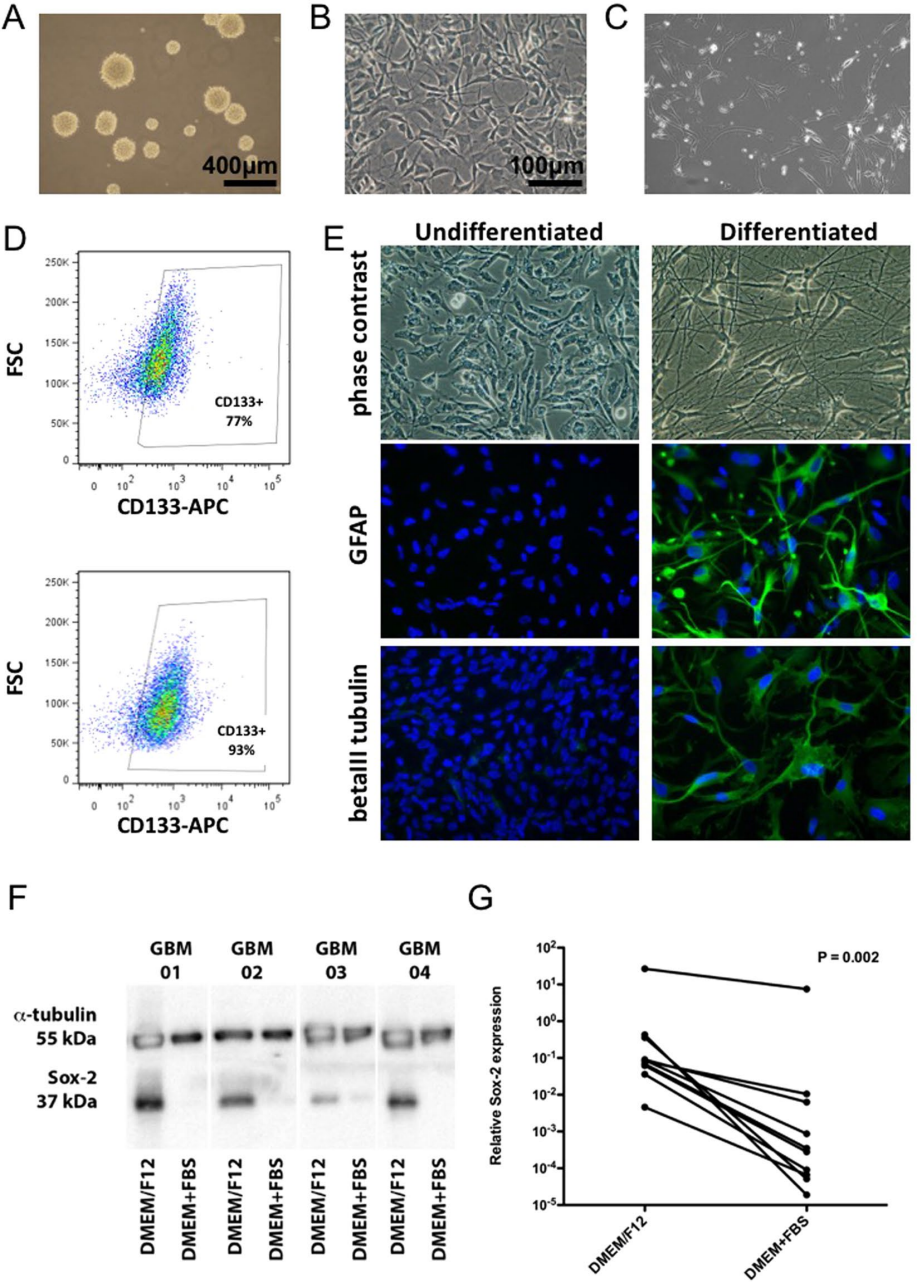
Characterization of the primary GBM cell cultures propagated in serum-free and serum containing media. (**A**) Cells growing in serum-free medium as gliomaspheres, (**B**) cells growing in serum-free medium on laminin, (**C**) adherent cell growing in serum containing medium, (**D**) detection of CD133 in two independent serum-free medium cultures, (**E**) differentiation of serum-free medium cultured cells induced by 10% fetal calf serum, (**F**) western blot analysis of Sox-2 and α-tubulin and (**G**) qRT-PCR of Sox-2 expression in GBM cells propagated in serum-free (DMEM/F12) and serum containing (DMEM + FBS) media. The P value signifies the statistical significance of the difference between the paired primary cell lines as assessed by the Wilcoxon paired test.

We successfully derived paired primary cell cultures from several GBMs (8 men and 2 women; median age 64 years - min 52, max 78 years), which were propagated in both defined serum-free medium favoring the expansion of GSCs and in medium supplemented with 10% FBS (non-stem cells). The cells cultured in serum-free medium initially formed gliomaspheres (Fig. 1A) and were subsequently propagated on laminin or geltrex (Fig. 1B). The matched paired primary cell cultures propagated in serum containing media grew adherently (Fig. 1C). The majority of the GSC cultures exhibited CD133 expression as determined by flow cytometry (Fig. 1D) and could undergo differentiation into GFAP and beta III tubulin expressing cells when transferred into serum containing media (Fig. 1E). All of the paired GBM cell cultures were IDH1/2 wild-type, their characteristics are summarized in Supplementary Table S1.

Western blot (Fig. 1F) and qRT-PCR (P = 0.002; Wilcoxon paired test) (Fig. 1G) analyses revealed substantially higher Sox-2 expression in the cells cultured in serum-free conditions in comparison to the cells derived from the same patient sample propagated in serum containing medium. The expression of nestin, another stem cell marker, correlated with Sox-2 expression (r = 0.7955; P < 0.0001) and there was a trend for higher nestin expression in the GBM primary cells derived in serum-free conditions (P = 0.065; Wilcoxon paired test) (see Supplementary Figure S1).

**Figure 2:**
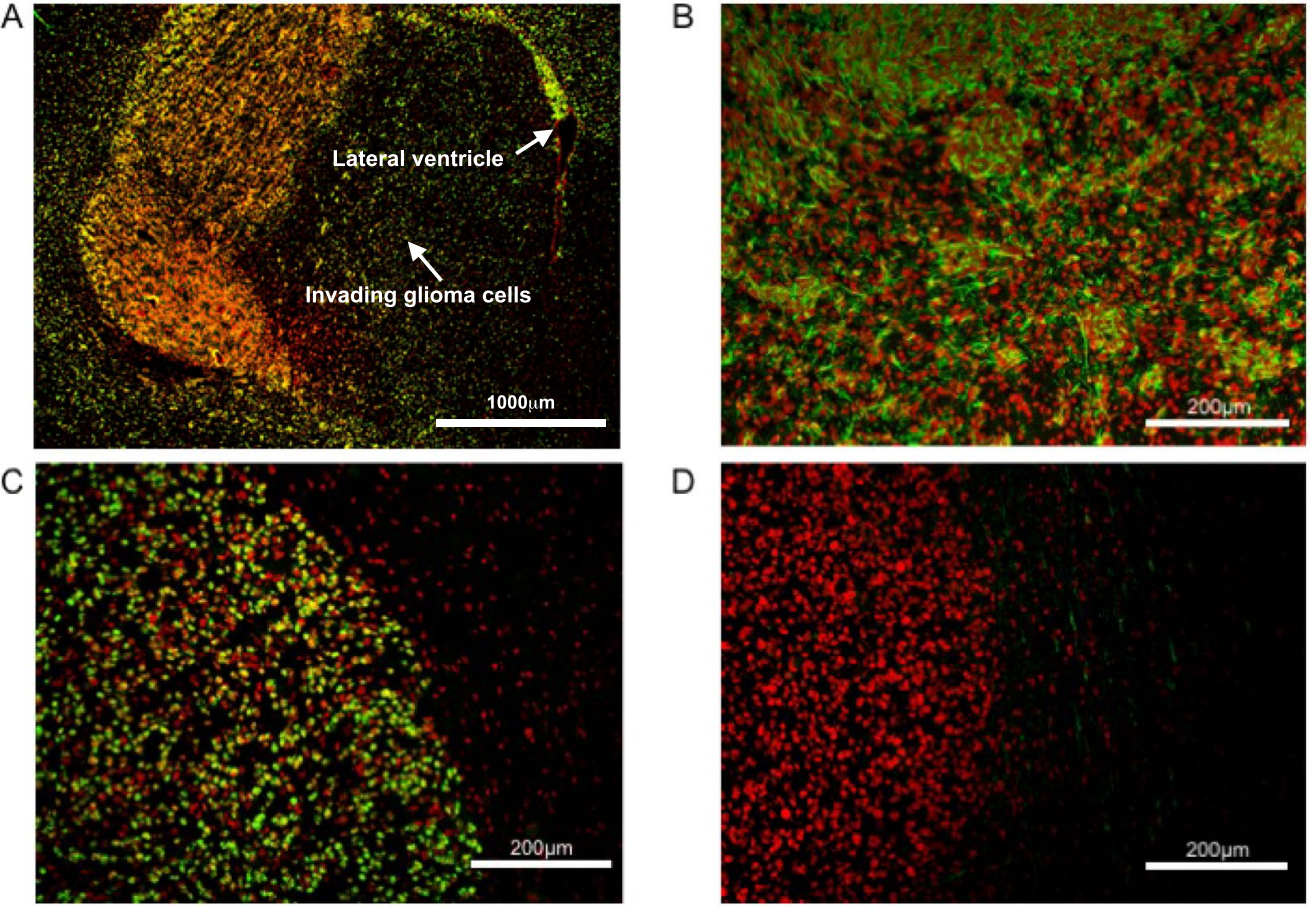
Characteristics of orthotopic xenotransplants derived from primary GBM cell cultures propagated in serum-free (**A**,**B**) and serum containing (**C**,**D**) media. (**A** and **C**) Detection of human glioma cells by an antibody against human nuclei (in green). (**B** and **D**) Detection of GFAP expression (in green). ToPro3 was used for nuclear counterstaining (red).

The large majority of the cultures propagated in serum-free as well as serum containing media were tumorigenic in immunodeficient mice (7/8 and 6/8 cultures, respectively). Nevertheless, the paired GBM cell cultures formed xenograft tumors with distinct features. The tumors derived from the glioma primary cell cultures propagated in serum containing media were characteristically well demarcated and GFAP negative (Fig. 2C,D). In contrast, the paired cultures derived in serum-free conditions typically produced GFAP positive tumors with single cell infiltration into the surrounding brain tissue including the contralateral hemisphere, white matter tracts and tropism towards the periventricular regions (Fig. 2A,B).

In summary, we verified that the cell cultures derived in serum-free conditions have typical characteristics of glioma stem-like cells (GSCs).

### MicroRNAs differentially expressed in the paired GSC and non-stem glioblastoma cell cultures

To identify a set of miRNAs characteristic for glioma stem-like cells we performed a genome-wide expression profiling of 2578 human miRNAs in the 10 paired GSC and non-stem GBM cell cultures derived in serum-free and serum supplemented medium, respectively. LIMMA analysis for paired samples revealed 431 significantly deregulated miRNAs in the GSCs in comparison with the non-stem GBM cells (P < 0.05) (see Supplementary Table S2). 51 miRNAs were deregulated at a significance level below 0.001 (25 miRNAs were upregulated and 26 miRNAs were downregulated). Importantly, among the 51 most deregulated miRNAs expression of 23 miRNAs correlated with Sox-2 expression at a significance level lower than 0.001, and 14 miRNAs correlated with both Sox-2 (P < 0.001) and nestin expression (P < 0.05) (Table 1). These data strongly suggest that several of the identified miRNAs are closely linked to the stemness of the glioma cell lines cultured in serum-free media.

**Table 1:** MiRNAs correlating with Sox-2 and nestin expression in paired primary GBM cell cultures.

	miRNA	Sox-2		Nestin	
		Spearman r	P value	Spearman r	P value
Negative correlation	miR-3195	−0.85	<10^-5^	−0.60	0.006
	miR-3141	−0.83	<10^-5^	−0.47	0.036
	miR-4656	−0.81	<10^-4^	−0.51	0.023
	miR-100-5p	−0.79	<10^-4^	−0.39	NS
	miR-4739	−0.77	<10^-3^	−0.42	NS
	miR-3180	−0.75	<10^-3^	−0.55	0.013
	miR-1260b	−0.75	<10^-3^	−0.46	0.043
	miR-1233-5p	−0.74	<10^-3^	−0.49	0.029
	miR-4674	−0.73	<10^-3^	−0.54	0.015
	miR-328-5p	−0.73	<10^-3^	−0.48	0.032
	miR-378h	−0.72	<10^-3^	−0.48	0.034
	miR-4505	−0.71	<10^-3^	−0.46	0.045
	miR-5787	−0.71	<10^-3^	−0.47	0.036
	miR-1207-5p	−0.70	<10^-3^	−0.37	NS
Positive correlation	miR-345-5p	0.82	<10^-5^	0.57	0.011
	miR-1180-3p	0.78	<10^-4^	0.45	0.048
	miR-9-3p	0.76	<10^-3^	0.40	NS
	miR-124-3p	0.75	<10^-3^	0.34	NS
	miR-106b-3p	0.73	<10^-3^	0.42	NS
	miR-1301-3p	0.73	<10^-3^	0.41	NS
	miR-130b-3p	0.71	<10^-3^	0.49	0.029
	miR-93-3p	0.70	<10^-3^	0.37	NS
	miR-106b-5p	0.70	<10^-3^	0.32	NS

**Figure 3:**
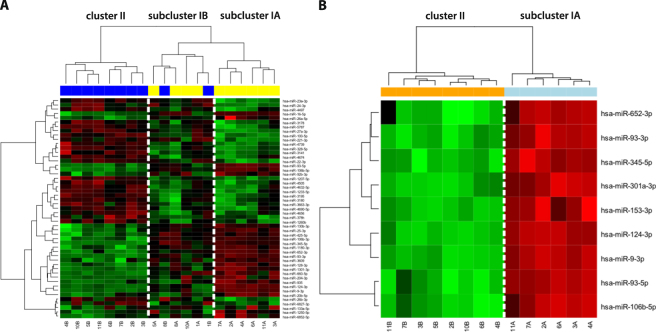
Hierarchical clustergram discriminating paired primary GSC (yellow) and non-stem glioblastoma cell cultures (blue) propagated in serum-free and serum containing medium, respectively. (**A**) Based on 51 differentially (P < 0.001) expressed miRNAs, (**B**)Based on 9 miRNAs differentially (P < 0.0001) expressed in GSC and non-stem cell cultures contained in subclusters IA and II, respectively. A gradient of green and red colors is used in the heatmap (green color indicates lower expression whereas red color indicates higher expression of individual miRNAs in analyzed samples).

Cluster analysis based on the 51 most differentially expressed miRNAs correctly classified all GSC and 80% of the non-stem cell cultures (Fig. 3A). This analysis also revealed that the main cluster I containing all GSC samples was divided into two subclusters. Subcluster IA was exclusively composed of GSC cultures and the pattern of miRNA expression was more distinct from serum cultures contained in cluster II. All analyzed cultures in subcluster IA were tumorigenic and exhibited pronounced multilineage differentiation. Subcluster IB, which contained the remaining four serum-free derived GSC cultures, also included two serum derived non-stem cell cultures; moreover, the serum-free derived cultures in this subcluster exhibited only little differentiation when exposed to 10% serum and one of them did not form tumors in immunodeficient mice. Collectively, these data suggest somewhat less pronounced stemness characteristics of GSC cultures in subcluster IB (Table 2, Supplementary Table S1). Statistical analysis comparing only cell cultures from subcluster IA, which exhibited more pronounced stemness characteristics, and cluster II samples containing the non-stem cell cultures revealed nine miRNAs (miR-9-3p, miR-93-3p, miR-93-5p, miR-106b-5p, miR-124-3p, miR-153-3p, miR-301a-3p, miR-345-5p, and miR-652-3p), which were all upregulated in GSCs at a significance level below 0.0001 (Fig. 3B). Expression of all these miRNAs positively and statistically significantly correlated with Sox2 expression suggesting their close association with the stem cell-like phenotype of the GSCs.

**Table 2:** Characteristics of the clusters identified based on the 51 most differentially expressed miRNAs.

Cluster	No. of GSC cultures	No. of non-stem cell cultures	Multilineage differentiation^‡^	Tumorigenicity
IA	6	0	4/4**	5/5
IB	4	2	0/3	2/3 (GSC) 1/2 (non-stem cells)
II	0	8	n.d.	5/6

^‡^Number of cell cultures exhibiting pronounced differentiation in serum containing media/number of analyzed cell cultures, **P < 0.05, Pearson’s chi-squared test compared to cluster IB, n.d. - not determined.

### MiRNAs differentially expressed in GSCs are associated with survival of GBM patients

**Figure 4:**
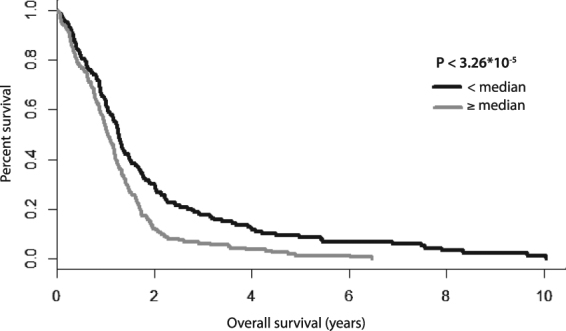
Kaplan-Meier survival curves estimating OS in GBM patients from the TCGA data set according to the 7-miRNA based Risk Score.

To further support the potential importance of these miRNAs in GBM, we analyzed their relation to overall survival (OS) using the TCGA dataset comprising 485 GBM patients for whom OS and miRNA expression profiles were available. Seven out of nine of the miRNAs most differentially expressed between the GSC cluster IA and non-stem cell cluster II (Fig. 3B, miR-9-3p, miR-93-5p, miR-106b-5p, miR-153-3p, miR-301a-3p, miR-345-5p, and miR-652-3p) were represented in the TCGA dataset. First, we performed Z-score transformation on expression levels across all GBM samples for each of the aforementioned seven miRNAs; then, the seven-miRNA signature was used to calculate the Risk Score for each patient based on a linear combination of the miRNA expression level weighted by the regression coefficient derived from the multivariate Cox regression analysis^22,23^ as follows: risk score = 0.08270698 * hsa-miR-652 + 0.15074626 * hsa-miR-345 − 0.11310809 * hsa-miR-301 − 0.15450420 * hsa-miR-153 + 0.09238838 * hsa-miR-9* − 0.18565873 * hsa-miR-93 − 0.04894894 * hsa-miR-106b. This composite miRNA Risk Score was a statistically significant prognostic factor in the univariate Cox regression analysis (HR = 2.718; 95% CI (1.814−4.073), P < 1.26 * 10^−6^). Correspondingly, using the median value of the miRNA Risk Score as the threshold, GBM patients could be divided into a high-risk and a low-risk group. Kaplan–Meier analysis confirmed that OS of the high-risk patients was significantly lower in comparison with low-risk patients (P < 3.26 * 10^−5^, log-rank test) (Fig. 4). We further tested the prognostic power of the seven-miRNA signature with respect to the IDH1 mutation status in a subset comprising 296 GBM patients for whom exome somatic mutation data were available. The univariate Cox regression analysis revealed that the seven-miRNA signature predicted OS in these patients with higher statistical significance (P = 1.064 * 10^−4^; HR = 2.718; 95% CI (1.642–4.501)) in comparison with IDH1 status (P = 3.3 * 10^−3^; HR = 0.3147; 95% CI (0.1393–0.7109)). These results were underscored using the multivariate Cox regression analysis (P = 6.53 * 10^−4^; HR = 2.442; 95% CI (1.461–4.080) for the seven-miRNA signature and P = 1.67*10^−2^; HR = 0.367; 95% CI (0.162–0.834) for IDH1 status). The whole model based on the seven-miRNA signature and IDH1 status predicted OS in GBM patients with a P value 4.24 * 10^−5^ (see Supplementary Table S3). Importantly, the seven-miRNA signature was able to predict OS in both IDH1 wild-type (n = 280) and IDH1 mutated (n = 16) GBM patients (P < 1.01 * 10^−3^ and P < 4.62 * 10^−2^, respectively; log-rank test) (see Supplementary Figure S2).

## Discussion

Glioblastoma multiforme (GBM), the most common malignant primary brain tumor arising from glial cells, is associated with fatal prognosis caused not only by its localization in the central nervous system, but especially by the high invasiveness and resistance to conventional therapies. This biological behavior is associated with the cellular and molecular heterogeneity, which is characteristic for this disease^24,25^. Recent studies have suggested that GBM is driven and maintained by a subpopulation of clonogenic cells called glioblastoma stem-like cells (GSCs), which seem to play a crucial role in GBM biology^26,27^. These cells also contribute to GBM chemoradioresistance through the activation of DNA damage checkpoint responses and the increase in DNA repair capacity^28,29^. The small non-coding microRNAs (miRNAs) playing an important role in the posttranscriptional regulation of gene expression have been previously described in association with GBM initiation, progression, and resistance to therapy as well as with the maintenance of glioma stem-like cells^30^.

In this study, we firstly successfully derived and characterized paired GBM cell cultures from several GBMs, which were propagated either under defined serum-free conditions, or in serum containing medium. These differing cell culture conditions most likely lead to the isolation of distinct cell subpopulations from the original tumor. Consistently with the literature, the paired cell lines displayed profound biological differences^31^. Glioma cells cultured in serum-free conditions frequently expressed CD133, although as described by others^6^ the quantity was variable in individual cell lines. The serum-free medium cultured cells also showed the potential to form gliomaspheres and differentiate into GFAP and beta III tubulin positive cells. In comparison with the paired glioma cultures derived in serum containing medium, the serum-free medium derived cells expressed significantly more Sox-2, the stemness marker crucial for the tumorigenicity of GSCs^32^, on both mRNA and protein levels. As previously reported^31^, the serum-free cultured cells characteristically formed highly infiltrative tumors when implanted into immunodeficient mice.

Having established that the cell lines cultured under serum-free conditions used in this study exhibit features typical of GSC, the paired GSC and non-stem cell cultures were utilized to uncover the miRNA expression pattern specific for GSC. Using global miRNA expression analysis, we revealed 51 most differentially expressed miRNAs. These miRNAs were able to classify the cell cultures into non-stem cell cluster II and two GSC subclusters IA and IB (Fig. 3A). Analysis of these two GSC subclusters showed that the first of them (IA) consisted of cultures with more pronounced GSC features compared to the second subcluster (IB) containing among others also two non-stem cell cultures. Subsequent analysis focusing on the miRNAs most differentially expressed between the GSC cluster IA and the non-stem cell cluster II highlighted nine miRNAs (miR-9-3p, miR-93-3p, miR-93-5p, miR-106b-5p, miR-124-3p, miR-153-3p, miR-301a-3p, miR-345-5p, and miR-652-3p) that were strongly upregulated in GSCs. Several of these miRNAs were previously described to be associated with the regulation of the stemness maintenance as well as with the biological behavior of GBM and survival of patients. MiR-9-3p (referred to as miR-9*) and its hairpin counterpart miR-9-5p (referred to as miR-9), which was also upregulated in GSCs though with lower statistical significance (p < 3.3 * 10^−3^; log2 FC = 2.1), seem to be specifically expressed in the brain^33,34^, are evolutionary conserved from insects to human and are involved in vertebrate neural development^35–37^. Their activities are probably carried out through the effect on the Notch signaling pathway, especially by the targeting of Notch2 and the transcription factor Hes1, resulting in an enhanced differentiation and proliferation of neural stem cells (NSCs)^38,39^. These data seem to be somewhat contradictory to our findings as we observed higher expression of both miRNAs in GSCs. The explanation may lie in the mutual regulation of miR-9/9* and Notch signaling. It was demonstrated that expression levels of miR-9/9* depend on the activation status of Notch signaling. While Notch inhibits differentiation of NSCs, it also induces miR-9/9*^38^. Moreover, Hes1 expression oscillates with a period of 2–3 hours in NSCs and this oscillation is important not only for cell differentiation but also for proliferation as sustained Hes1 expression inhibits both processes^39,40^. Thus, it can be assumed that miR-9/9* expression levels vary in time to allow cell proliferation. It also seems that the control mechanisms in GSCs are different from those in NSCs. In line with our findings, Schraivogel *et al*. reported that both miRNAs were highly abundant in CD133+ GSCs and their inhibition led to the reduced neurosphere formation and stimulated cell differentiation^41^. Finally, the higher levels of miR-9/9* hairpin counterparts in GSCs could also contribute to the increased resistance of these cells to the conventional therapy. Munoz *et al*. recently showed that CD133+ GSCs expressed greater levels of miR-9 which led to the activation of the SHH/PTCH1/MDR1 axis. This axis has been shown to impart TMZ resistance. In the case of the CD133+ cells, the resistance is not acquired but seems to be inherent^42^.

Interestingly, miR-9 and miR-9* seem to be functionally linked with the miR-124, another most differentially expressed miRNA identified in our study. Staahl *et al*. published that mitotic exit in neurogenesis is inter alia partially driven by these three matured miRNAs^43^. Another study described the synergistic effect of miR-9 and miR-124 on the strong suppression of the GTP-binding protein Rap2a and consequent promotion of neuronal differentiation of NSCs and dendritic branching of differentiated neurons^44^. A very similar effect of the overexpression of miR-9/9* and miR-124 on the self-renewal and differentiation was observed by Roese-Koerner *et al*. in neuroepithelial-like stem cells derived from human pluripotent stem cells^40^.

MiR-106b-5p, miR-93-5p as well as miR-93-3p are members of the same miRNA gene cluster miR-106b~25 and it is thus not surprising that these miRNAs were jointly upregulated. Interestingly, this cluster seems to be linked to the biology of stem cells. Serum induced differentiation of GSCs was previously demonstrated to decrease the expression levels of miRNAs which belong to this cluster^18^ and Brett *et al*. showed that the expression of the entire miR-106b~25 cluster in adult mouse neuronal stem/progenitor cells increases their ability to generate new neurons^45^. In the CD44+ gastric cancer stem-like cells, the entire cluster was significantly upregulated and inhibition of miR-106b led to a decreased self-renewal capacity and cell invasiveness through the suppression of the TGF-β/Smad signaling pathway^46^.

Only few studies suggesting a direct link between the other miRNAs identified in our study and stem cell biology are available so far. Chang *et al*. described miR-345 to be enriched in mesenchymal stem cells found in Wharton’s jelly matrix of human umbilical cord which were able to transdifferentiate into neuronal lineage cells^47^. The miR-301 family has been recently shown to be the direct target of the SFRS2 splicing factor, an OCT4 regulated gene required for the pluripotency in human pluripotent stem cells^48^. Stappert *et al*. demonstrated that miR-153 contributes to the shift of long-term self-renewing neuroepithelial-like stem cells from self-renewal to neuronal differentiation^49^. Similarly, Tezcan *et al*. also demonstrated that miR-153 overexpression reduced tumorigenic capacity of GSCs by targeting the Nrf-2/GPx1/ROS pathway^50^. In contrast with these data, we observed higher expression of both miR-153 and the members of the miR-301 family (miR-301a-3p, miR-130b-3p, and miR-130a-3p) in GSCs. Unfortunately, there are no studies which could help to explain these discrepancies. However, it seems that the stem cell-like state is dependent on many interacting molecules in feedback loops mutually balancing one another over time.

The Risk Score utilized in this study proved that the set of the identified miRNAs is associated with GBM prognosis independently of IDH1 mutation status, further suggesting their involvement in the disease pathogenesis. Higher tissue levels of miR-652, miR-345 and miR-9* positively contributed to increased values of the risk score and thus worse prognosis; nevertheless, miR-301, miR-153, miR-93 and miR-106b which were also upregulated in GSCs were in fact negative contributors. The explanation for these results is at present speculative but may involve the following aspects. Firstly, the relation between GSCs and survival is somewhat unclear as various studies failed to show a correlation between the GSC quantity assessed by CD133, nestin or CD15 staining and survival^51,52^. Further, Pallini *et al*. observed that the percentage of CD133-positive cells somewhat paradoxically correlated with longer survival in recurrent glioblastoma, likely due to the higher presence of normal neural stem cells with possible antitumor properties, which may also apply to some newly diagnosed tumors^53^. Another study described that the CD133-low GBMs showed more invasive growth and gene expression profiles characteristic of mesenchymal or proliferative subtypes, whereas the CD133-high GBMs showed features of cortical and well-demarcated tumors and gene expressions typical of proneuronal subtypes. Moreover, in contrast to various reports claiming that only CD133-positive GBM cells can initiate tumor formation *in vivo*, Joo *et al*. showed that CD133-negative cells also possess tumor-initiating potential^54^. It should be further noted that CSCs are rather quiescent and slow-cycling, and some of the identified miRNAs may be regulators of this dormant state^55^. Last but not least, non-transformed stromal cells may contribute to the tissue levels of miRNAs and the function of the particular miRNAs may be different in these cells compared to glioma cells.

Taken together, we identified a set of miRNAs that are differentially expressed in GSCs as compared to non-stem glioblastoma cells, several of which correlated with the expression of the stem cell markers Sox-2 and nestin. Our findings thus suggest that a complex set of miRNAs is involved in the regulation of the stem-like characteristics in glioblastoma. Moreover, a set of the most differentially expressed miRNAs correlated with the survival of GBM patients independently of the IDH1 status indicating that they might be prognostic markers and possibly new therapeutic targets.

## Material and Methods

### GBM samples and primary cell cultures

The 10 paired primary GBM cell cultures were derived from fresh tumor tissues samples obtained from GBM patients who underwent surgically resection at the Departments of Neurosurgery of the Hospital Na Homolce in Prague, University Hospital Brno, and University Hospital Ostrava. Study has been approved by the local Ethical Board at Hospital Na Homolce in Prague, University Hospital Brno, and University Hospital Ostrava. Written informed consent was obtained from all patients included in the study. All experiments were performed in accordance with relevant guidelines and regulations.

The fresh tissue sample was enzymatically dissociated with TrypLE (ThermoFisher Scientific) for 20 min at 37 °C with agitation or using the Papain Dissociation System (Worthington) according to manufacturer’s instructions. Single cell suspensions were seeded into 25 cm^2^ tissue culture flasks (Techno Plastic Products AG) and cultured in either Dulbecco’s modified essential medium supplemented with 10% FBS, 1% Glutamax (both ThermoFisher Scientific), 100 U/mL penicillin and 100 μg/mL streptomycin, sodium pyruvate and non-essential amino acids (all GE Healthcare), or in DMEM/F12 containing bFGF 20 ng/mL, EGF 20 ng/mL (both PeproTech), B27-supplement 1:50 (ThermoFisher Scientific), 1% Glutamax, 100 U/mL penicillin and 100 μg/mL streptomycin. After 1–3 weeks, adherent cells, which covered more than 2/3 of the culture flask in DMEM, were passaged using Trypsin-EDTA solution (Sigma-Aldrich). After approximately the same time, tumor spheres formed in DMEM/F12, and these were dissociated using Accutase (Sigma-Aldrich) and up and down pipetting and then passaged. Cells that initially formed spheres were dissociated and transferred to laminin (Sigma-Aldrich) or Geltrex (ThermoFisher Scientific) coated culture flasks and propagated as monolayer cultures^56,57^. For the subsequent analyses, early passage cultures were used.

### qRT-PCR quantification

Complementary DNA (cDNA) was synthesized from 1000 ng small RNA enriched total RNA using the High Capacity cDNA Reverse Transcription Kit (ThermoFisher Scientific) according to the manufacturer’s protocol. qRT-PCR was performed using the LightCycler 480 Instrument II (Roche) in accordance with the standard TaqMan manufacturer’s protocol using TaqMan Gene Expression Assays (GAPDH #Hs03929097_g1, SOX2 #Hs01053049_s1, NES #Hs00707120_s1; ThermoFisher Scientific). The data were evaluated using the second derivative maximum method with the arithmetic baseline adjustment. All qRT-PCR reactions were run in triplicate and average Cp and SD values were calculated. Relative expression levels were determined by the 2^−ΔCp^ method, where ΔCp was calculated as follows: ΔCp = Cp (gene of interest) − Cp (GAPDH).

### Western blot analysis

Cell pellets were lysed with RIPA buffer (Sigma-Aldrich). Protein concentration was measured using a DC Protein Assay (Bio-Rad), samples were diluted with RIPA buffer to attain the same concentration of total protein and boiled for 10 min with the Laemmli sample buffer. Proteins (15 μg per well) were separated on 10% SDS-PAGE gels, and electrophoretically transferred to the polyvinylidene difluoride (PVDF) membrane (Merck Millipore). The membranes were blocked with 5% nonfat milk in PBS with 0.1% Tween 20 (PBS-T), then incubated either with an anti-Sox2 rabbit mAb or anti-alpha/beta-tubulin rabbit mAb (No. 3579 and 2148, respectively; both Cell Signaling Technology) diluted 1:1000 in blocking solution at 4 °C overnight. Subsequently, the membranes were incubated with anti-rabbit IgG antibody HRP conjugate (No. 7074, Cell Signaling Technology) diluted 1:2500 (60 min/RT). Each step was followed by washes in PBS-T. ECL-Plus detection was performed according to the manufacturer’s instructions (Amersham).

### Flow cytometry

Accutase (Sigma-Aldrich) was used to harvest adherent cells and dissociate gliomaspheres. The cell suspension was fixed with 2% paraformaldehyde for 1 hour at 4 °C, permeabilized (Intracellular Staining Permeabilization Wash Buffer, Biolegend) and stained using an anti-CD133 APC conjugated antibody (Miltenyi Biotec).

### Differentiation of stem-like cell cultures, immunocytochemistry

To induce differentiation, 13 × 10^3^ cells per cm^2^ were plated on geltrex coated coverslips and cultured in differentiation medium (DMEM/F12, 10% FBS, 1% Glutamax, 100 μg/mL Streptomycin and 100 U/mL Penicillin G). The medium was exchanged every 2–3 days for 10–14 days. The coverslips were subsequently fixed with 4% paraformaldehyde (10 minutes at room temperature) and stained overnight at 4 °C using the antibodies against GFAP (GF-01, Exbio, 1:200) and beta III tubulin (Exbio, 1:250).

### Orthotopic xenotransplantation glioma model, immunohistochemistry

The experimental use of animals was approved by The Commission for Animal Welfare of the First Faculty of Medicine of the Charles University in Prague and The Ministry of Education, Youth and Sports of the Czech Republic according to the animal protection laws. Generation of xenotrasplants was performed as described previously^58^. 5 × 10^5^ cells in 5 µL of serum free medium were injected into 6–10 week old male NOD.129S7(B6)-Rag1tm1Mom/J mice (The Jackson Laboratory) with a Hamilton syringe 1.2 mm anterior from the bregma and 2.5 mm lateral from the midline to a depth of 3 mm using a stereotactic device (Stoelting Co.). Immunohistochemistry was performed on 10 μm thick frozen sections using antibodies against human nuclei (Chemicon, 1:500) and GFAP (GF-01, Exbio, 1:200) as described^59^.

### MiRNA microarray analysis

Small RNA enriched total RNA was isolated using the mirVana miRNA Isolation Kit (ThermoFisher Scientific). Nucleic acid concentrations and purities were controlled by UV spectrophotometry using Nanodrop ND-1000 (Thermo Scientific). To assess miRNA expression in Sox-2 high- and low-expressing GBM cells, the samples were analyzed with Affymetrix GeneChip miRNA 4.0 arrays (Affymetrix) containing 5607 probe sets for human small RNAs. Out of these probe sets the 2578 probe sets of human mature miRNAs were filtered. All steps of the procedure were performed according to the Affymetrix standardized protocol for miRNA 4.0 arrays. Intensity values for each probe cell (.cel file) were calculated using Affymetrix GeneChip Command Console (AGCC). Quality control of the microarray was performed with the Affymetrix miRNA QC Tool, version 1.1.1.0.

### Microarray expression data analysis

All data were pre-processed and further analyzed by the software packages included in the R/Bioconductor^60^. Pre-processing was performed by the RMA method with default parameters as implemented in the Bioconductor package oligo^61^. All data were log2-transformed. To identify differentially expressed miRNAs, the LIMMA approach^62^ for paired samples was applied with additional Benjamini-Hochberg correction of P values. To determine the correlation between miRNA and Sox-2 or nestin expression, the Spearman rank correlation coefficient was used.

### IDH1/2 mutation status analysis

DNA was extracted using QIAamp DNA Mini Kit (Qiagen, Germany) according to manufacturer’s instructions. Fragments of 254 bp and 293 bp lengths spanning the sequences encoding the catalytic domains of IDH1 including codon 132 and IDH2 including codon 172, respectively were amplified using 12,5 pmol each of the primers: IDH1-F ACCAAATGGCACCATACGA, IDH1-R TTCATACCTTGCTTAATGGGTGT, IDH2-F GCTGCAGTGGGACCACTATT, and IDH2-R TGTGGCCTTGTACTGCAGAG (primer sequences according to Hartmann *et al*., 2009). PCR was performed using standard buffer conditions, 50–250 ng of DNA input and Taq DNA Polymerase (Invitrogen, USA). PCR consisted of 35 cycles with denaturing at 95 °C for 30 s, annealing at 56 °C for 1 min and extension at 72 °C for 1 min in a total volume of 25 μl. Two microliters of the PCR amplification product were subjected to sequencing using the BigDye Terminator v3.1 Sequencing Kit (Applied Biosystems, USA). Twenty-five cycles were performed employing 0,5 μl of 10 μM primer IDH1-F ACCAAATGGCACCATACGA or IDH2-R TGTGGCCTTGTACTGCAGAG, with denaturing at 96 °C for 30 s, annealing at 50 °C for 15 s and extension at 60 °C for 4 min in a total volume of 10 μl. Sequences were determined using the sequencer (ABI 3500 Genetic Analyzer, Applied Biosystems) and the Mutation surveyor V4.0.9 software (SoftGenetics, USA).

### Survival analysis

The relationship between overall survival and expression levels of the selected miRNAs was analyzed on The Cancer Genome Atlas (TCGA) data set (485 GBM patients)^63^. To assess the miRNAs that were identified in this study for survival prediction, a Risk Score formula for predicting survival was developed based on a linear combination of the miRNA expression level weighted by the regression coefficient derived from the multivariate Cox regression analysis^22,23^. Patients with high Risk Score are expected to have poor survival. Cox proportional hazards regression analysis was performed to assess the contribution of the miRNA signature to survival prediction^64^. Patients were further stratified into a high-risk group and a low-risk group according to the Risk Score (cutoff value median) and survival was analyzed using the Kaplan-Meier method. Subsequently, both univariate and multivariate Cox regression analyses including seven-miRNA signature and IDH1 status were performed on the data subset comprising 296 GBM patients for whom exome somatic mutation data were available.

## Electronic supplementary material

Supplementary Information
