# Supplementary material for: Identification of microRNAs differentially expressed in glioblastoma stem-like cells and their association with patient survival
Source: Sci Rep. 2018 Feb 12;8:2836. doi: 10.1038/s41598-018-20929-6 (PMC5809429; doi:10.1038/s41598-018-20929-6)
Supplement: Supplementary file 1 — Supplementary Information [file 41598_2018_20929_MOESM1_ESM.pdf]

Identification of microRNAs differentially expressed in glioblastoma stem-like cells and their association with patient survival

Jiri Sana, Petr Busek, Pavel Fadrus, Andrej Besse, Lenka Radova, Marek Vecera, Stefan Reguli, Lucie Sromova, Marek Hilser, Radim Lipina, Radek Lakomy, Leos Kren, Martin Smrcka, Aleksi Sedo, Ondrej Slaby

Supplementary Figure S1

qRT-PCR quantification of nestin expression in GBM cells propagated in serum free (DMEM/F12) and serum containing (DMEM+FBS) media. **The p value signifies the statistical significance of the difference between the paired primary cell lines as assessed by the Wilcoxon paired test.**

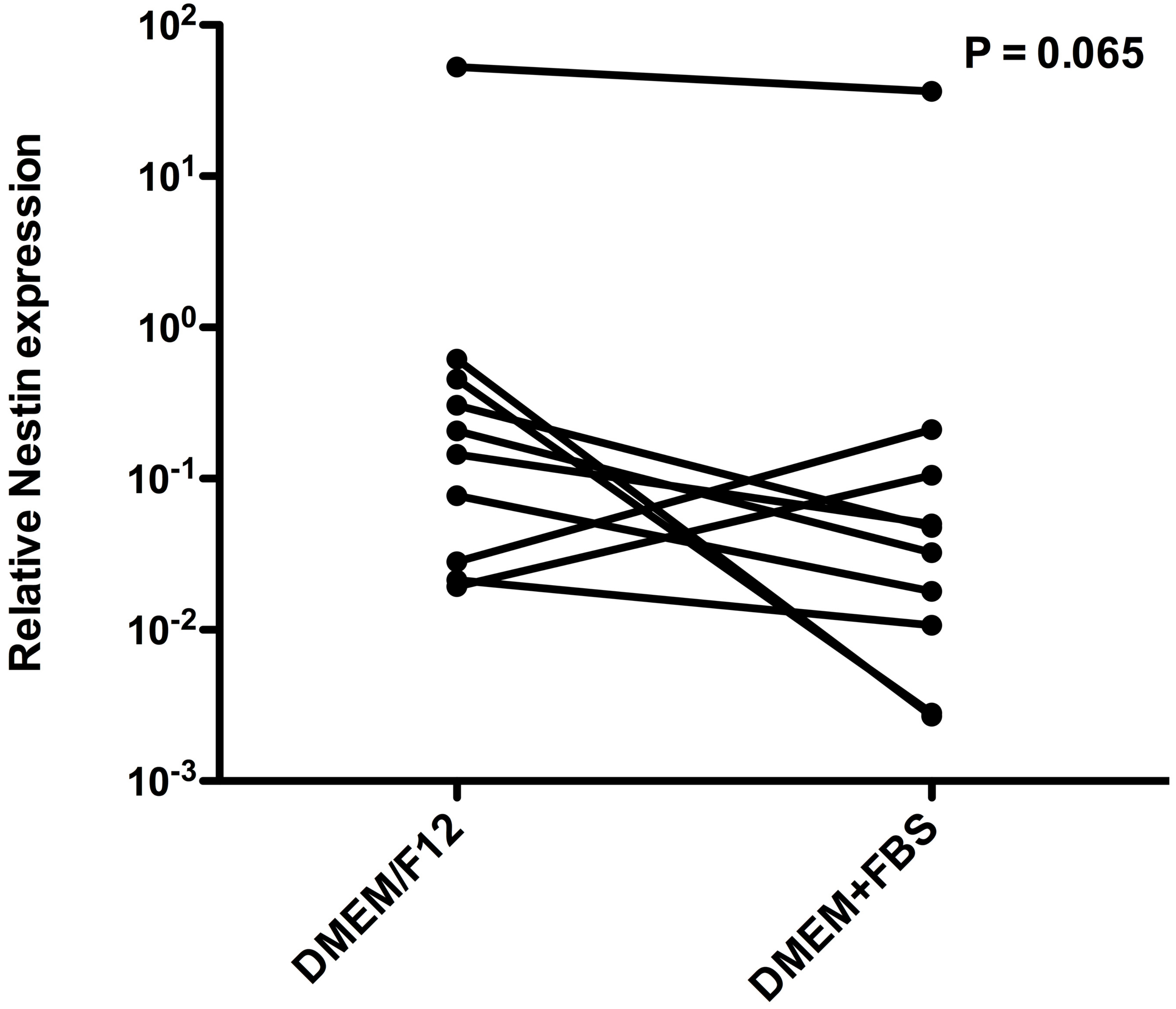

Supplementary Figure S2 **Kaplan-Meier survival curves estimating OS in GBM patients from the TCGA data set according to the 7-miRNA based Risk Score. A) IDH1 wild-type patients (n = 280), B) IDH1 mutated patients (n = 16).**

A

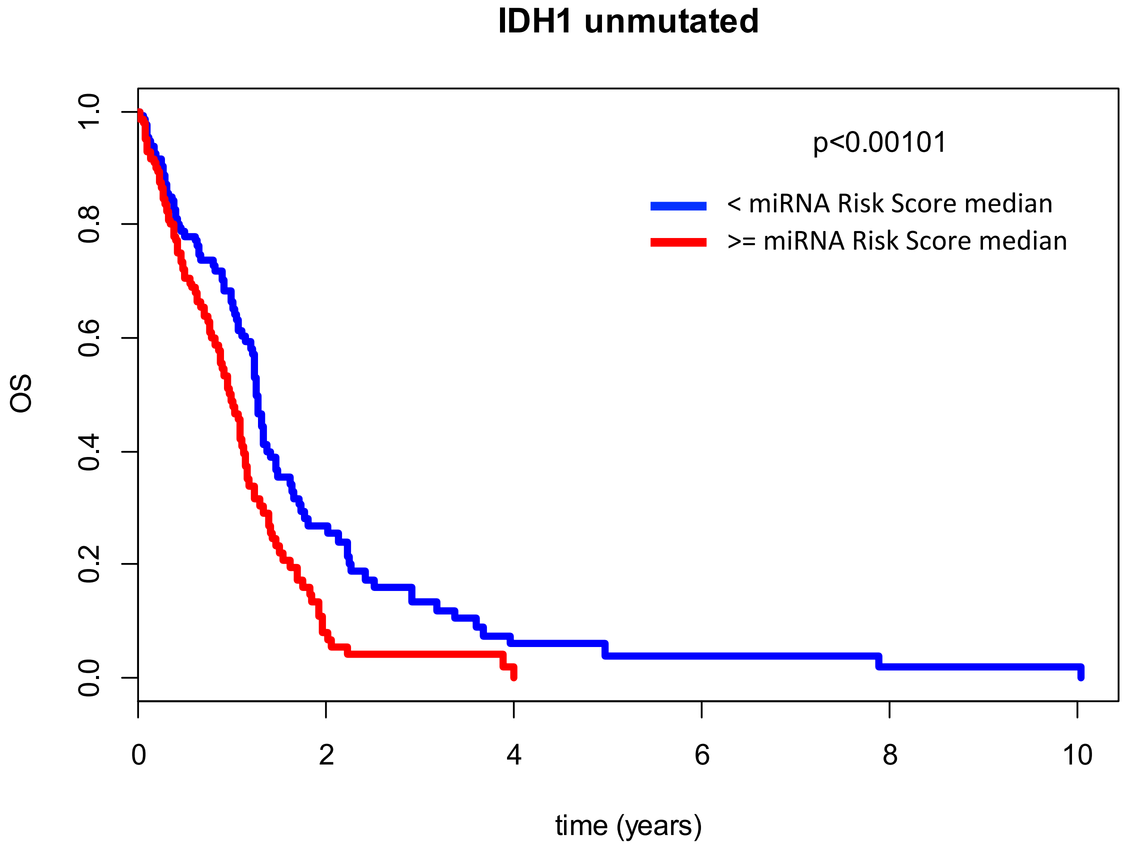

HR=1.61
95%CI (1.21 - 2.15)

B

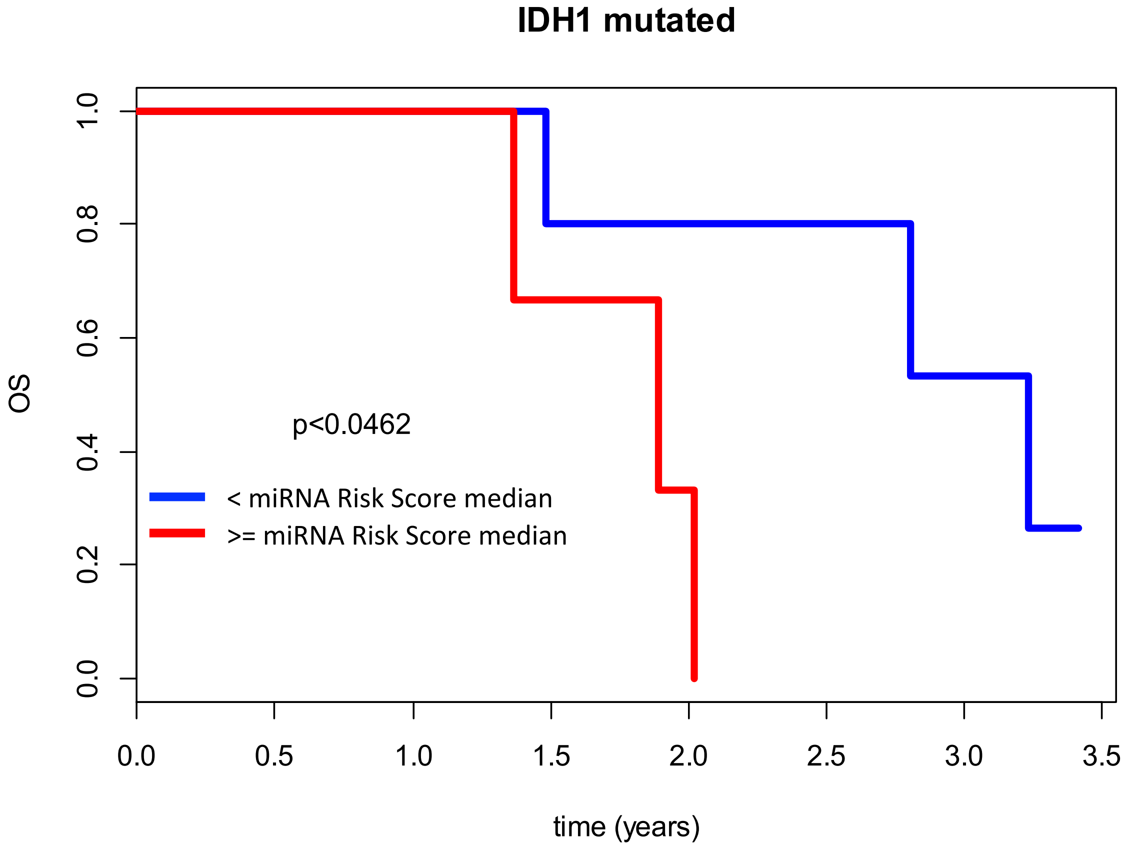

HR=7.4
95%CI (0.75 - 72.9)

Supplementary Table S1

Characteristics of the primary cell cultures used in the study

Cell culture code	miRNA cluster	Cell culture type	Multilineage differentiation of GSC cultures	Tumorigenicity in nude mice	Percentage of CD133+ cells (flow cytometry)	Age	Sex	IDH1	IDH2
1A	IB	GSC culture	No	Nontumorigenic	79	73	F	wt	wt
2A	IA	GSC culture	Yes	Tumorigenic	99	63	M	wt	wt
3A	IA	GSC culture	n.d.	Tumorigenic	76	52	M	wt	wt
4A	IA	GSC culture	Yes	Tumorigenic	93	72	M	wt	wt
5A	IB	GSC culture	No	Tumorigenic	60	62	M	wt	wt
6A	IA	GSC culture	Yes	Tumorigenic	97	78	M	wt	wt
7A	IA	GSC culture	Yes	Tumorigenic	85	58	F	wt	wt
8A	IB	GSC culture	No	Tumorigenic	87	63	M	wt	wt
10A	IB	GSC culture	n.d.	n.d.	n.d.	67	M	wt	wt
11A	IA	GSC culture	n.d.	n.d.	n.d.	65	M	wt	wt
1B	IB	Non-stem cell culture		Nontumorigenic	n.d.	*73*	*F*	*wt*	*wt*
2B	II	Non-stem cell culture		Tumorigenic	33	*63*	*M*	*wt*	*wt*
3B	II	Non-stem cell culture		Tumorigenic	8	*52*	*M*	*wt*	*wt*
4B	II	Non-stem cell culture		Tumorigenic	n.d.	*72*	*M*	*wt*	*wt*
5B	II	Non-stem cell culture		Nontumorigenic	n.d.	*62*	*M*	*wt*	*wt*

6B	II	Non-stem cell culture		Tumorigenic	n.d.	*78*	*M*	*wt*	*wt*
7B	II	Non-stem cell culture		Tumorigenic	3	*58*	*F*	*wt*	*wt*
8B	IB	Non-stem cell culture		Tumorigenic	n.d.	*63*	*M*	*wt*	*wt*
10B	II	Non-stem cell culture		n.d.	n.d.	*67*	*M*	*wt*	*wt*
11B	II	Non-stem cell culture		n.d.	n.d.	*65*	*M*	*wt*	*wt*

n.d.- not determined
wt- wild-type

Supplementary Table S2

Significantly deregulated miRNAs in glioma stem-like cell (GSC) cultures in comparison with non-stem glioblastoma cell cultures (P < 0.05; LIMMA Analysis)

microRNA	log2FC	Average Expression	P value
hsa-miR-124-3p	5,5	4,20	1,5E-05
hsa-miR-9-3p	4,8	3,87	1,8E-05
hsa-miR-4497	-1,8	10,97	2,8E-05
hsa-miR-1260b	-1,4	4,52	3,3E-05
hsa-miR-3195	-2,4	5,28	6,8E-05
hsa-miR-4656	-1,0	3,58	1,0E-04
hsa-miR-3178	-1,5	9,54	1,4E-04
hsa-miR-652-3p	2,5	3,41	1,6E-04
hsa-miR-378h	-1,0	3,41	2,1E-04
hsa-miR-204-3p	2,9	2,66	2,2E-04
hsa-miR-106b-5p	2,2	8,82	2,4E-04
hsa-miR-1233-5p	-1,1	5,45	2,5E-04
hsa-miR-4632-5p	-1,4	5,48	2,8E-04
hsa-miR-16-5p	1,2	10,52	3,1E-04
hsa-miR-1207-5p	-1,1	6,21	3,2E-04
hsa-miR-4505	-1,4	6,05	3,3E-04
hsa-miR-22-3p	-1,8	8,42	3,4E-04
hsa-miR-92b-3p	2,3	8,01	3,6E-04
hsa-miR-3663-3p	-1,9	4,73	3,7E-04
hsa-miR-23a-3p	-1,0	11,33	4,0E-04
hsa-miR-20b-5p	3,0	4,83	4,0E-04
hsa-miR-93-5p	2,1	9,07	4,1E-04
hsa-miR-93-3p	3,1	3,48	4,4E-04
hsa-miR-24-3p	-0,6	10,58	4,6E-04
hsa-miR-6852-5p	0,4	0,85	5,1E-04
hsa-miR-5787	-1,2	9,50	5,8E-04
hsa-miR-25-3p	2,4	6,13	5,9E-04
hsa-miR-130b-3p	2,6	6,62	6,1E-04
hsa-miR-6827-3p	-0,3	0,80	6,3E-04
hsa-miR-3180	-1,8	4,93	6,3E-04
hsa-miR-1250-5p	0,4	1,00	7,2E-04
hsa-miR-4674	-1,3	7,87	7,5E-04
hsa-miR-935	3,0	3,19	7,7E-04
hsa-miR-660-5p	1,9	2,48	8,1E-04
hsa-miR-345-5p	3,0	4,57	8,1E-04
hsa-miR-27a-3p	-1,1	9,03	8,4E-04

hsa-miR-4690-5p	-1,1	4,45	8,4E-04
hsa-miR-1180-3p	2,1	3,64	8,5E-04
hsa-miR-4739	-1,3	7,42	8,5E-04
hsa-miR-128-3p	2,2	2,80	8,6E-04
hsa-miR-3609	1,7	3,71	8,7E-04
hsa-miR-100-5p	-1,3	10,21	9,1E-04
hsa-miR-1301-3p	1,9	2,26	9,1E-04
hsa-miR-133a-5p	0,2	0,86	9,1E-04
hsa-miR-425-5p	1,3	6,47	9,3E-04
hsa-miR-26a-5p	1,2	10,49	9,4E-04
hsa-miR-26b-3p	-0,3	0,84	9,5E-04
hsa-miR-328-5p	-1,2	7,36	9,6E-04
hsa-miR-106b-3p	2,8	4,91	9,7E-04
hsa-miR-3141	-0,9	7,25	9,7E-04
hsa-miR-221-3p	-1,3	9,72	9,9E-04
hsa-miR-455-5p	1,4	1,87	1,1E-03
hsa-miR-149-5p	3,6	5,05	1,1E-03
hsa-miR-6865-5p	-1,2	3,22	1,2E-03
hsa-miR-3620-5p	-1,0	6,16	1,2E-03
hsa-miR-574-3p	-2,5	6,23	1,2E-03
hsa-miR-6771-5p	-1,2	5,87	1,3E-03
hsa-miR-193b-3p	-1,3	6,12	1,3E-03
hsa-miR-346	2,1	2,66	1,3E-03
hsa-miR-550a-5p	0,6	1,71	1,3E-03
hsa-miR-4634	-1,5	4,64	1,4E-03
hsa-miR-6765-5p	-1,4	7,49	1,4E-03
hsa-miR-762	-1,3	9,20	1,5E-03
hsa-miR-550a-3p	1,6	2,33	1,5E-03
hsa-miR-25-5p	1,4	3,17	1,5E-03
hsa-miR-328-3p	1,7	2,92	1,5E-03
hsa-miR-331-3p	2,1	2,41	1,6E-03
hsa-miR-4523	-0,3	0,94	1,6E-03
hsa-miR-188-5p	0,5	1,72	1,6E-03
hsa-miR-6743-5p	-1,1	7,25	1,6E-03
hsa-miR-6858-5p	-1,0	7,50	1,7E-03
hsa-miR-503-5p	1,4	4,74	1,7E-03
hsa-miR-1271-5p	2,0	4,09	1,8E-03
hsa-miR-320a	1,0	10,28	1,8E-03
hsa-miR-4281	-1,4	7,61	1,8E-03
hsa-miR-5189-5p	-1,2	3,49	1,9E-03
hsa-miR-103a-3p	1,8	10,00	1,9E-03
hsa-miR-23a-5p	-1,7	2,71	1,9E-03
hsa-miR-542-5p	2,0	2,82	1,9E-03
hsa-miR-4745-5p	-1,3	8,65	2,0E-03
hsa-miR-4749-5p	-1,1	4,83	2,0E-03
hsa-miR-6126	-1,9	6,45	2,0E-03
hsa-miR-324-5p	2,4	4,95	2,0E-03
hsa-miR-107	1,7	9,54	2,0E-03

hsa-miR-30c-5p	1,2	6,85	2,1E-03
hsa-miR-342-3p	2,1	7,14	2,1E-03
hsa-miR-3591-3p	-0,4	0,89	2,2E-03
hsa-miR-6816-5p	-1,2	8,74	2,2E-03
hsa-miR-421	2,5	2,76	2,3E-03
hsa-miR-6085	-0,9	5,82	2,3E-03
hsa-miR-6752-5p	-1,2	6,21	2,4E-03
hsa-miR-885-5p	1,7	2,21	2,5E-03
hsa-miR-6778-5p	-1,0	4,75	2,6E-03
hsa-miR-153-3p	1,6	1,91	2,6E-03
hsa-miR-4492	-1,1	6,87	2,6E-03
hsa-miR-663a	-1,1	8,80	2,6E-03
hsa-miR-8075	-1,2	8,28	2,6E-03
hsa-miR-320c	0,9	9,94	2,7E-03
hsa-miR-106a-5p	1,1	8,92	2,7E-03
hsa-miR-7109-5p	-0,8	3,00	2,8E-03
hsa-miR-200c-5p	0,3	0,90	2,8E-03
hsa-miR-3621	-1,1	7,14	2,8E-03
hsa-miR-6084	-0,3	1,18	2,8E-03
hsa-miR-6732-5p	-1,1	7,86	2,9E-03
hsa-miR-181a-5p	1,5	9,24	2,9E-03
hsa-miR-4763-3p	-1,2	7,66	2,9E-03
hsa-miR-6775-5p	-0,7	7,20	3,0E-03
hsa-miR-6756-5p	-0,9	5,95	3,0E-03
hsa-miR-17-5p	1,0	9,11	3,0E-03
hsa-miR-1231	-1,8	4,99	3,1E-03
hsa-miR-495-3p	1,6	2,09	3,1E-03
hsa-miR-8071	1,4	2,37	3,1E-03
hsa-miR-551b-3p	1,7	1,80	3,2E-03
hsa-miR-301a-3p	2,2	2,19	3,3E-03
hsa-miR-9-5p	2,1	1,87	3,3E-03
hsa-miR-134-3p	0,7	1,45	3,3E-03
hsa-miR-320b	0,9	10,14	3,3E-03
hsa-miR-6786-5p	-1,1	8,96	3,4E-03
hsa-miR-3180-3p	-1,4	5,38	3,4E-03
hsa-miR-1184	-0,9	3,64	3,5E-03
hsa-miR-193a-5p	-2,0	5,53	3,5E-03
hsa-miR-181a-3p	1,8	2,62	3,5E-03
hsa-miR-6805-5p	-1,1	8,21	3,7E-03
hsa-miR-411-3p	0,6	1,71	3,7E-03
hsa-miR-4508	-1,0	9,33	3,8E-03
hsa-miR-339-3p	1,5	3,70	3,9E-03
hsa-miR-6880-5p	-0,9	3,64	3,9E-03
hsa-miR-195-5p	2,0	6,19	4,0E-03
hsa-miR-1228-5p	-1,2	8,30	4,0E-03
hsa-miR-4707-5p	-1,2	7,92	4,3E-03
hsa-miR-1909-3p	-1,4	4,64	4,4E-03
hsa-miR-200c-3p	0,5	1,24	4,4E-03

hsa-miR-339-5p	1,6	4,54	4,4E-03
hsa-miR-4459	-0,8	6,54	4,4E-03
hsa-miR-210-3p	-1,3	6,03	4,5E-03
hsa-miR-6511b-5p	-0,9	4,82	4,6E-03
hsa-miR-320d	0,9	7,89	4,7E-03
hsa-miR-29a-3p	-1,5	8,35	4,8E-03
hsa-miR-99b-3p	1,1	2,73	4,8E-03
hsa-miR-6803-5p	-1,0	8,31	5,0E-03
hsa-miR-182-5p	4,2	5,22	5,1E-03
hsa-miR-15b-5p	0,8	8,04	5,1E-03
hsa-miR-543	2,0	3,03	5,1E-03
hsa-miR-297	1,0	1,59	5,2E-03
hsa-miR-10b-5p	1,8	3,01	5,3E-03
hsa-miR-1976	-0,3	1,04	5,4E-03
hsa-miR-30d-5p	1,7	4,70	5,4E-03
hsa-miR-361-5p	0,6	8,24	5,4E-03
hsa-miR-324-3p	1,9	4,17	5,4E-03
hsa-miR-130a-3p	1,8	6,02	5,4E-03
hsa-miR-18b-5p	1,9	2,12	5,6E-03
hsa-miR-6791-5p	-1,1	8,22	5,7E-03
hsa-miR-514a-3p	-0,3	0,76	5,7E-03
hsa-miR-497-5p	2,3	3,40	5,8E-03
hsa-miR-4485	-1,3	4,00	5,8E-03
hsa-miR-4486	-1,2	5,29	5,9E-03
hsa-miR-1237-5p	-1,1	9,78	5,9E-03
hsa-miR-4687-3p	-1,0	8,75	6,1E-03
hsa-miR-6749-5p	-0,7	5,66	6,1E-03
hsa-miR-487a-3p	2,3	3,62	6,2E-03
hsa-miR-4463	-1,0	6,07	6,4E-03
hsa-miR-6894-5p	-0,6	1,86	6,4E-03
hsa-miR-4298	-1,0	7,37	6,4E-03
hsa-miR-1469	-1,1	9,47	6,4E-03
hsa-miR-183-5p	2,7	2,76	6,5E-03
hsa-miR-941	0,7	1,52	6,5E-03
hsa-miR-377-3p	0,4	1,26	6,6E-03
hsa-miR-6787-5p	-1,0	4,72	6,7E-03
hsa-miR-6789-5p	-1,0	8,00	6,8E-03
hsa-miR-145-5p	-3,3	4,63	6,9E-03
hsa-miR-2277-5p	-0,8	3,97	6,9E-03
hsa-miR-760	0,6	1,27	7,0E-03
hsa-miR-4442	-0,2	1,06	7,1E-03
hsa-miR-6722-3p	-0,9	6,67	7,2E-03
hsa-miR-222-3p	-0,9	10,40	7,3E-03
hsa-miR-3940-5p	-1,1	8,93	7,4E-03
hsa-miR-769-5p	1,0	1,90	7,6E-03
hsa-miR-181c-3p	0,9	1,39	7,6E-03
hsa-miR-195-3p	0,5	1,11	7,7E-03
hsa-miR-4784	-0,3	1,24	7,8E-03

hsa-miR-30a-3p	1,6	3,26	8,0E-03
hsa-miR-6850-5p	-1,0	8,29	8,0E-03
hsa-miR-30a-5p	2,4	5,36	8,0E-03
hsa-miR-3196	-0,9	10,19	8,1E-03
hsa-miR-4665-5p	-1,3	4,33	8,1E-03
hsa-miR-18a-5p	1,6	5,52	8,1E-03
hsa-miR-4532	-1,2	5,96	8,2E-03
hsa-miR-3656	-1,0	9,16	8,3E-03
hsa-miR-342-5p	0,5	1,39	8,4E-03
hsa-miR-655-3p	-0,2	1,10	8,4E-03
hsa-miR-619-5p	1,9	4,73	8,5E-03
hsa-miR-6808-5p	-0,6	2,65	8,5E-03
hsa-miR-6132	-1,0	3,00	8,6E-03
hsa-miR-215-5p	-0,2	0,91	8,7E-03
hsa-miR-1915-5p	0,3	0,99	9,1E-03
hsa-miR-3672	-0,3	0,95	9,2E-03
hsa-miR-6807-5p	0,9	1,36	9,3E-03
hsa-miR-7854-3p	0,2	1,04	9,3E-03
hsa-miR-550a-3-5p	0,6	1,53	9,4E-03
hsa-miR-2909	-0,2	0,82	9,4E-03
hsa-miR-127-5p	1,0	2,09	9,4E-03
hsa-miR-204-5p	2,0	2,02	9,5E-03
hsa-miR-6800-5p	-1,1	8,64	9,7E-03
hsa-miR-3200-3p	1,0	1,71	9,8E-03
hsa-miR-181d-5p	1,8	3,35	9,9E-03
hsa-miR-3667-3p	-0,5	1,09	1,0E-02
hsa-miR-34a-5p	-0,8	7,01	1,0E-02
hsa-miR-532-5p	1,8	4,90	1,0E-02
hsa-miR-887-3p	0,9	1,48	1,0E-02
hsa-miR-376a-3p	1,4	2,38	1,0E-02
hsa-miR-4797-5p	0,3	1,12	1,1E-02
hsa-miR-4709-3p	0,3	0,99	1,1E-02
hsa-miR-4638-3p	0,4	1,09	1,1E-02
hsa-miR-409-5p	1,8	3,24	1,1E-02
hsa-miR-149-3p	-1,0	8,80	1,1E-02
hsa-miR-4649-5p	-1,3	5,11	1,1E-02
hsa-miR-519d-5p	-0,3	0,83	1,1E-02
hsa-miR-485-3p	2,1	3,78	1,1E-02
hsa-miR-6794-5p	-0,9	5,22	1,1E-02
hsa-miR-4785	-0,6	1,90	1,1E-02
hsa-miR-758-5p	0,4	1,19	1,1E-02
hsa-miR-323a-3p	1,1	1,40	1,1E-02
hsa-miR-181b-5p	1,8	7,96	1,1E-02
hsa-miR-127-3p	3,2	6,32	1,2E-02
hsa-miR-4286	0,8	1,97	1,2E-02
hsa-miR-589-3p	0,7	1,77	1,2E-02
hsa-miR-488-5p	-0,2	0,90	1,2E-02
hsa-miR-1185-2-3p	2,1	3,59	1,2E-02

hsa-miR-2861	-1,1	9,28	1,2E-02
hsa-miR-1225-5p	-1,3	3,09	1,2E-02
hsa-miR-6879-5p	-0,7	4,02	1,2E-02
hsa-miR-362-5p	1,9	3,14	1,2E-02
hsa-miR-1245a	-0,2	0,90	1,2E-02
hsa-miR-433-3p	1,9	2,83	1,2E-02
hsa-miR-425-3p	1,1	1,90	1,2E-02
hsa-miR-4270	-0,7	6,75	1,2E-02
hsa-miR-194-3p	0,2	0,94	1,2E-02
hsa-miR-4507	-0,9	4,74	1,2E-02
hsa-miR-424-3p	1,6	4,36	1,3E-02
hsa-miR-194-5p	1,1	2,90	1,3E-02
hsa-miR-2110	-0,6	1,59	1,3E-02
hsa-miR-181c-5p	1,4	2,24	1,3E-02
hsa-miR-4516	-1,2	9,62	1,4E-02
hsa-miR-6724-5p	-1,0	9,11	1,4E-02
hsa-miR-320e	1,4	5,14	1,4E-02
hsa-miR-548g-3p	-0,2	0,84	1,4E-02
hsa-miR-8072	-0,9	9,29	1,4E-02
hsa-miR-1275	-1,1	4,58	1,4E-02
hsa-miR-19b-3p	0,7	7,31	1,5E-02
hsa-miR-597-3p	-0,2	0,88	1,5E-02
hsa-miR-183-3p	1,3	1,80	1,5E-02
hsa-miR-6769b-5p	-0,4	4,19	1,5E-02
hsa-miR-1227-5p	-1,1	6,77	1,5E-02
hsa-miR-363-3p	2,2	2,01	1,5E-02
hsa-miR-148b-5p	-0,3	0,91	1,5E-02
hsa-miR-4734	-1,0	8,36	1,6E-02
hsa-miR-545-5p	-0,3	0,75	1,6E-02
hsa-miR-494-3p	2,6	5,79	1,6E-02
hsa-miR-4327	-0,3	1,05	1,6E-02
hsa-miR-6879-3p	0,2	0,96	1,6E-02
hsa-miR-7108-5p	-0,9	8,54	1,6E-02
hsa-miR-5001-5p	-0,9	7,66	1,6E-02
hsa-miR-6821-5p	-1,0	7,51	1,6E-02
hsa-miR-330-3p	0,6	1,69	1,7E-02
hsa-miR-23b-3p	-0,5	9,90	1,7E-02
hsa-miR-766-5p	0,3	0,94	1,7E-02
hsa-let-7d-5p	0,8	8,84	1,7E-02
hsa-miR-3185	-1,2	7,16	1,7E-02
hsa-miR-3200-5p	0,5	1,46	1,7E-02
hsa-miR-5009-3p	-0,3	0,89	1,7E-02
hsa-miR-4429	1,2	5,84	1,8E-02
hsa-miR-4695-5p	-0,8	6,03	1,8E-02
hsa-miR-4750-5p	-0,9	4,21	1,8E-02
hsa-miR-4511	-0,2	0,87	1,8E-02
hsa-miR-548o-3p	-0,2	0,82	1,8E-02
hsa-miR-638	-0,9	9,98	1,8E-02

hsa-miR-30b-5p	1,5	4,95	1,9E-02
hsa-miR-4488	-1,0	10,22	1,9E-02
hsa-miR-4689	-1,0	4,13	1,9E-02
hsa-miR-378a-3p	-1,4	6,33	1,9E-02
hsa-miR-1908-5p	-0,9	9,43	2,0E-02
hsa-miR-6877-3p	1,0	1,42	2,0E-02
hsa-miR-154-5p	0,9	1,99	2,0E-02
hsa-miR-4440	1,1	2,06	2,0E-02
hsa-miR-939-3p	-0,2	0,87	2,0E-02
hsa-miR-758-3p	0,9	1,90	2,0E-02
hsa-miR-138-1-3p	-1,7	2,92	2,0E-02
hsa-miR-584-5p	0,6	1,26	2,1E-02
hsa-miR-4793-3p	0,9	2,01	2,1E-02
hsa-miR-6727-5p	-0,9	10,18	2,1E-02
hsa-miR-219a-1-3p	0,2	0,91	2,1E-02
hsa-miR-3124-5p	-0,8	2,38	2,1E-02
hsa-miR-6812-5p	-0,8	5,84	2,2E-02
hsa-miR-7641	-1,0	4,85	2,2E-02
hsa-let-7d-3p	0,6	1,58	2,2E-02
hsa-miR-3119	-0,2	0,79	2,2E-02
hsa-miR-20b-3p	0,8	1,16	2,2E-02
hsa-miR-6845-5p	-0,7	1,63	2,2E-02
hsa-miR-6826-3p	-0,2	1,02	2,3E-02
hsa-miR-4721	-0,9	2,24	2,3E-02
hsa-miR-15a-5p	1,5	5,32	2,3E-02
hsa-miR-410-3p	0,4	1,45	2,3E-02
hsa-miR-4498	-0,8	3,40	2,3E-02
hsa-miR-3689a-5p	-0,3	0,84	2,3E-02
hsa-miR-3689b-5p	-0,3	0,84	2,3E-02
hsa-miR-3689e	-0,3	0,84	2,3E-02
hsa-miR-376b-3p	0,5	1,34	2,3E-02
hsa-miR-1185-1-3p	1,8	3,99	2,4E-02
hsa-miR-4501	-0,2	0,92	2,5E-02
hsa-miR-31-3p	-0,7	1,40	2,5E-02
hsa-miR-338-5p	0,3	1,09	2,5E-02
hsa-let-7c-5p	0,5	10,60	2,5E-02
hsa-miR-6828-5p	-0,2	0,84	2,5E-02
hsa-miR-154-3p	0,7	1,40	2,6E-02
hsa-miR-423-3p	0,5	7,35	2,6E-02
hsa-miR-125b-2-3p	1,2	3,00	2,7E-02
hsa-miR-500a-5p	1,3	3,14	2,7E-02
hsa-miR-591	-0,2	0,85	2,7E-02
hsa-miR-6754-5p	-0,4	1,24	2,7E-02
hsa-miR-374b-3p	-0,2	0,89	2,7E-02
hsa-miR-3679-3p	-0,2	1,13	2,7E-02
hsa-miR-3675-5p	0,2	0,94	2,7E-02
hsa-miR-7847-3p	-0,9	8,12	2,7E-02
hsa-miR-615-3p	1,1	2,08	2,7E-02

hsa-miR-6869-5p	-0,9	10,53	2,7E-02
hsa-miR-3960	-0,6	11,82	2,8E-02
hsa-miR-4640-5p	-0,6	2,26	2,8E-02
hsa-miR-4668-5p	-0,7	10,04	2,8E-02
hsa-miR-6788-5p	0,1	0,92	2,8E-02
hsa-miR-3907	0,4	1,15	2,8E-02
hsa-miR-1268b	-0,6	4,30	2,8E-02
hsa-miR-329-3p	1,0	1,73	2,9E-02
hsa-miR-3671	-0,3	0,86	2,9E-02
hsa-miR-4664-3p	0,2	0,92	2,9E-02
hsa-miR-187-5p	-0,3	1,20	2,9E-02
hsa-miR-134-5p	2,1	4,88	3,0E-02
hsa-miR-138-5p	-2,6	6,09	3,0E-02
hsa-miR-4466	-0,8	10,49	3,0E-02
hsa-miR-150-3p	-0,9	1,96	3,0E-02
hsa-miR-3187-3p	-0,8	3,65	3,0E-02
hsa-miR-548ay-5p	-0,2	0,90	3,1E-02
hsa-miR-6502-5p	-0,2	0,96	3,1E-02
hsa-miR-1268a	-0,7	4,67	3,1E-02
hsa-miR-370-5p	0,3	1,06	3,2E-02
hsa-miR-4502	0,5	1,27	3,2E-02
hsa-miR-379-5p	2,2	5,16	3,2E-02
hsa-miR-6729-5p	-0,7	10,36	3,2E-02
hsa-miR-21-3p	-0,8	2,29	3,2E-02
hsa-miR-374c-3p	-0,2	0,98	3,2E-02
hsa-miR-503-3p	0,4	0,91	3,3E-02
hsa-miR-191-5p	0,4	9,58	3,3E-02
hsa-miR-3685	0,2	1,06	3,3E-02
hsa-miR-409-3p	2,2	5,71	3,3E-02
hsa-miR-19a-3p	0,6	2,16	3,3E-02
hsa-miR-4481	0,4	1,53	3,3E-02
hsa-miR-148b-3p	1,0	1,53	3,4E-02
hsa-miR-192-5p	0,7	1,88	3,4E-02
hsa-miR-8062	-0,2	0,81	3,4E-02
hsa-miR-370-3p	1,7	3,85	3,4E-02
hsa-miR-4490	-0,4	1,23	3,4E-02
hsa-miR-18a-3p	0,6	1,33	3,4E-02
hsa-miR-99a-5p	0,9	8,02	3,4E-02
hsa-miR-3613-3p	-0,6	10,40	3,4E-02
hsa-miR-4677-5p	-0,2	0,82	3,5E-02
hsa-miR-148a-3p	1,1	2,13	3,5E-02
hsa-miR-6820-5p	-0,8	3,14	3,5E-02
hsa-miR-6750-5p	-0,6	4,30	3,5E-02
hsa-miR-3664-5p	-0,2	0,80	3,6E-02
hsa-miR-6502-3p	-0,2	0,85	3,6E-02
hsa-miR-7704	-0,8	11,26	3,6E-02
hsa-miR-199a-5p	-1,8	3,52	3,6E-02
hsa-miR-3940-3p	0,3	1,19	3,6E-02

hsa-miR-1297	-0,2	0,91	3,7E-02
hsa-miR-29b-2-5p	0,2	0,99	3,7E-02
hsa-miR-143-3p	-2,4	3,70	3,7E-02
hsa-miR-5188	-0,3	1,15	3,7E-02
hsa-miR-4484	-1,5	6,04	3,7E-02
hsa-miR-1913	-0,2	1,20	3,8E-02
hsa-miR-4671-3p	-0,2	0,80	3,8E-02
hsa-let-7g-5p	1,3	5,53	3,8E-02
hsa-miR-3925-5p	0,2	0,96	3,8E-02
hsa-miR-769-3p	0,6	1,29	3,8E-02
hsa-miR-6089	-0,7	11,38	3,9E-02
hsa-miR-4266	-0,2	0,87	3,9E-02
hsa-miR-4723-3p	-0,2	0,87	3,9E-02
hsa-miR-132-3p	1,5	4,99	4,0E-02
hsa-miR-4731-5p	-0,3	1,06	4,0E-02
hsa-miR-432-5p	1,9	4,53	4,0E-02
hsa-miR-604	-0,2	0,84	4,1E-02
hsa-miR-3131	-0,2	1,07	4,1E-02
hsa-miR-214-3p	-1,8	3,79	4,1E-02
hsa-miR-8059	-0,2	0,83	4,2E-02
hsa-miR-6798-5p	-0,9	4,69	4,2E-02
hsa-miR-596	-0,2	0,99	4,2E-02
hsa-miR-628-3p	0,2	1,03	4,2E-02
hsa-miR-410-5p	-0,2	0,93	4,2E-02
hsa-miR-500a-3p	1,2	4,40	4,3E-02
hsa-miR-937-5p	-0,7	5,28	4,3E-02
hsa-miR-126-3p	0,7	2,00	4,3E-02
hsa-miR-331-5p	0,4	1,10	4,3E-02
hsa-miR-1915-3p	-0,7	10,24	4,3E-02
hsa-miR-30b-3p	0,3	1,07	4,3E-02
hsa-miR-1245b-5p	-0,2	0,82	4,4E-02
hsa-miR-602	-0,9	3,85	4,4E-02
hsa-miR-1343-5p	-0,9	6,60	4,4E-02
hsa-miR-31-5p	-1,7	3,99	4,4E-02
hsa-miR-96-5p	0,7	1,31	4,5E-02
hsa-miR-6860	-0,6	2,71	4,5E-02
hsa-miR-766-3p	0,6	2,13	4,5E-02
hsa-miR-203b-5p	-0,2	0,81	4,5E-02
hsa-miR-6088	-0,7	10,12	4,5E-02
hsa-miR-744-5p	1,0	6,34	4,6E-02
hsa-miR-668-5p	-0,6	2,77	4,6E-02
hsa-miR-3074-3p	0,3	0,98	4,6E-02
hsa-miR-4651	-0,8	6,39	4,7E-02
hsa-miR-598-5p	0,4	0,88	4,7E-02
hsa-miR-550b-2-5p	0,4	1,23	4,8E-02
hsa-miR-20a-5p	0,7	8,20	4,8E-02
hsa-miR-6764-3p	-0,2	0,78	4,8E-02
hsa-miR-4467	-0,8	6,96	4,8E-02

hsa-miR-4462	0,4	1,57	4,9E-02
hsa-miR-135b-3p	0,3	0,87	4,9E-02
hsa-miR-3134	-0,2	1,03	4,9E-02
hsa-miR-532-3p	1,3	3,77	4,9E-02
hsa-miR-3646	-0,2	1,06	4,9E-02
hsa-miR-1252-5p	-0,2	0,95	4,9E-02
hsa-miR-17-3p	0,5	2,03	4,9E-02
hsa-miR-514b-3p	-0,3	0,79	4,9E-02
hsa-miR-496	0,2	0,97	4,9E-02
hsa-miR-7113-3p	-0,3	1,11	5,0E-02
hsa-miR-4305	-0,2	0,87	5,0E-02

Supplementary Table S3 **Univariate and multivariate Cox regression analyses of seven-miRNA signature and IDH1 status in relation to the OS in GBM patients (n = 296)**

	univariate					multivariate					
	coef	HR	95% HR		pvalue	coef	HR	95% HR		pvalue	model pvalue
seven-miRNA signature	1,00	2,72	1,64	4,50	**1,06E-04**	0,89	2,44	1,46	4,08	**6,53E-04**	**4,24E-05**
IDH1 status: mutated	-1,16	0,31	0,14	0,71	**3,30E-03**	-1,00	0,37	0,16	0,83	**1,67E-02**	
